# Activation of liver X receptors protects oligodendrocytes in CA3 of stress-induced mice

**DOI:** 10.3389/fphar.2022.936045

**Published:** 2022-07-25

**Authors:** Peilin Zhu, Jing Tang, Xin Liang, Yanmin Luo, Jin Wang, Yue Li, Kai Xiao, Jing Li, Yuhui Deng, Lin Jiang, Qian Xiao, Yingqiang Qi, Yuhan Xie, Hao Yang, Lin Zhu, Yong Tang, Chunxia Huang

**Affiliations:** ^1^ Department of Histology and Embryology, Chongqing Medical University, Chongqing, China; ^2^ Laboratory of Stem Cells and Tissue Engineering, Chongqing Medical University, Chongqing, China; ^3^ Department of Pathophysiology, Chongqing Medical University, Chongqing, China; ^4^ Department of Physiology, Chongqing Medical University, Chongqing, China; ^5^ Lab Teaching and Management Center, Chongqing Medical University, Chongqing, China; ^6^ Department of Radioactive Medicine, Chongqing Medical University, Chongqing, China; ^7^ Department of Electron Microscope, Institute of Life Science, Chongqing Medical University, Chongqing, China

**Keywords:** liver X receptors agonist, depression, chronic unpredictable stress, oligodendrocyte, CA3

## Abstract

Depression is a complex disorder that is associated with various structural abnormalities. Oligodendrocyte (OL) dysfunction is associated with the pathogenesis of depression and the promotion of hippocampal oligodendrocyte maturation and myelination could be a novel therapeutic strategy for ameliorating depressive behaviors. Recent studies have shown that activation of liver X receptors (LXRs) by GW3965 improves depressive phenotypes, but the effects of GW3965 on OL function and myelination in the hippocampus of depression remain relatively unclear. To address this issue, we investigated the effects of GW3965 on mature OL in the hippocampus and on the myelin sheaths of mice subjected to chronic unpredictable stress (CUS). Behavioral tests were performed to assess depressive behaviors. Then, the number of mature OLs (CC1^+^) in each hippocampal subregion was precisely quantified with immunohistochemical and stereological methods, and the density of newborn mature OLs (BrdU^+^/Olig2^+^/CC1^+^ cells) in each hippocampal subregion was quantified with immunofluorescence. In addition, myelin basic protein (MBP) staining intensity in the cornu ammonis 3 (CA3) region was assessed by using immunofluorescence. We found that both the number of CC1^+^ OLs and the density of BrdU^+^/Olig2^+^/CC1^+^ cells were obviously decreased in each hippocampal subregion of mice subjected to CUS, and 4 weeks of GW3965 treatment reversed these effects only in the CA3 region. Furthermore, the decreased MBP expression in the CA3 region of mice subjected to CUS was ameliorated by GW3965 treatment. Collectively, these results suggested that improvement of OL maturation and enhancement of myelination may be structural mechanisms underlying the antidepressant effects of LXR agonists.

## Introduction

Major depressive disorder (MDD) is a common lifelong mental disorder that is characterized by recurrent episodes (Malhi and Mann, 2018; Vos et al., 2020); MDD is a leading cause of mental health-related disease burden worldwide. Approximately 300 million people around the world suffer from depression (Herrman et al., 2019). Although there are many hypotheses regarding the pathogenesis of depression, no conclusive results have been reported (Kupfer, Frank and Phillips, 2012; Boku et al., 2018). In addition, several strategies, including antidepressant therapy, psychotherapy, and electroconvulsive therapy, have been evaluated as potential clinical treatments for depression. In many MDD patients, these treatments are effective, but some patients do not maintain symptom remission after clinical treatment (Fava, 2003). Therefore, more effective treatments, including new antidepressant drugs are urgently needed.

In recent years, the roles of pathological changes in oligodendrocytes (OLs) in the etiology of depression have attracted increasing attention (Wang et al., 2014; Zhou et al., 2021). OLs produce substances, including lipids and proteins, that are indispensable for the formation of the myelin sheath (Elbaz and Popko, 2019) which is fundamental to both excitatory and inhibitory neuron function and connectivity (Boda, 2021). Nugent et al. reported decreases in myelin-related protein expression and mRNA transcription in OLs in the ventrolateral prefrontal cortices of MDD patients (Nugent et al., 2019). Matthew D et al. also demonstrated that myelin loss occurs in the whole brains of depressive patients (Sacchet and Gotlib, 2017). These changes suggest that altered myelin may participate in the pathogenesis of depressive disorders. Cathomas et al. found that chronic social stress reduces the expression of OL-related genes in mice; these genes may therefore be therapeutic targets treating stress-induced depression in the future (Cathomas et al., 2019). Furthermore, Liu et al. showed that clemastine (an antimuscarinic compound) can promote adult myelination and OL differentiation in the medial prefrontal cortex (mPFC), which suggests that clemastine treatment might be a potential strategy for reversing depressive-like social behavior (Liu et al., 2016). Moreover, the hippocampus, which is known as the “emotional brain,” is responsible for the regulation of learning, memory and emotion. A large number of studies have shown that the hippocampus is a particularly important brain region that is affected by major depression, and it is obviously altered in depressed patients in response to treatment (MacQueen et al., 2003; MacQueen and Frodl, 2011; Bromis et al., 2018; Roddy et al., 2019; Nogovitsyn et al., 2020). Abnormalities in hippocampal structure and function are important factors that result in depression. Consistently, Tang provided evidence that the total volume of myelinated nerve fibers, total volume of myelin sheaths and total number of OLs in the hippocampus are significantly decreased in stressed mice (Tang et al., 2019). Taken together, these data suggest that oligodendroglia-targeted approaches may be novel therapeutic strategies for depressive disorders.

Liver X receptors (LXRs), including LXRα (*NR1H3*) and LXRβ (*NR1H2*), are members of the nuclear receptor (NR) superfamily and act as ligand-activated transcription factors (Gabbi, Warner and Gustafsson, 2009). LXRα is expressed predominantly in the liver and other organs related to lipid metabolism, whereas LXRβ is widely expressed in all tissues and is abundantly expressed in the central nervous system (Zhang, 2002). Interestingly, related studies have indicated that LXRβ-knockout mice exhibit anxiety-like behaviors (Tan et al., 2012; Yu et al., 2020) and that rats in which LXRβ is knocked down in the hippocampus by short hairpin RNA (*shRNA*) exhibit depressive-like behaviors; however, the LXR agonist GW3965 can improve depressive-like behaviors (Peng et al., 2018). These findings suggest that dysregulation of LXRs could be involved in the risk of depression. Previous studies have revealed that LXRβ regulates the maturation/myelination of OLs and the differentiation of OL progenitor cells (OPCs) by driving radial glial cells (RGCs) to become OPCs in the dorsal cortex (Xu et al., 2014). Mitro et al. showed that an LXR agonist (GW3965) can increase the gene expression of myelin basic protein (MBP), which is a protein believed to be important in the myelination of nerves in the central nervous system (CNS), in steroidal diabetes model rats (Mitro et al., 2012). Moreover, LXRs and their target genes (*ABCA1*, *ABCG1*, *ABCG4*, *APOE* and *LDLR*) are crucial for cholesterol homeostasis and play important roles in the demyelination and remyelination of the myelin sheaths formed by OLs (Zhang, 2002; Jakobsson et al., 2012; Nelissen et al., 2012). Recently, LXR target gene products have been shown to facilitate the efflux of lipids and cholesterol to support the remyelination of OLs (Berghoff et al., 2021). This evidence strongly indicates that LXRs are involved in OL differentiation and myelination. However, whether the positive influences of LXR agonists on OLs participate in the behavioral regulation of depression has not been reported.

In the present study, we treated male mice that were exposed to chronic unpredictable stress (CUS) with the LXR agonist GW3965 and used a sucrose preference test (SPT), forced swimming test (FST) and open field test (OFT) to examine the antidepressant effects of this agonist. Then, for the first time, we systematically quantified the numbers of mature OLs (CC1^+^ cells) in subregions (the cornu ammonis [CA] 1, CA3 and dentate gyrus [DG]) of the hippocampus in the mice in each group with immunohistochemical techniques and modern stereological methods. We also estimated the staining intensity in the hippocampal subregions of Olig2^+^/BrdU^+^ cells and CC1^+^/Olig2^+^/BrdU^+^ cells and the expression of MBP in the CA3 with immunofluorescence. Our results are expected to provide a structural basis and identify the therapeutic targets of LXR agonists in the treatment of depression.

## Materials and methods

### Experimental animals and groups

Adult male C57BL/6 mice aged 4–6 weeks were obtained from the Laboratory Animal Center of Chongqing Medical University. All the experimental animals were raised for 2 weeks and allowed to adapt to the new housing environment before any experiments were initiated. Approximately four to five animals were group-housed in each cage; the mice were housed under a 12-h/12-h light/dark cycle and at a stable room temperature (22 ± 2°C), and all of them had free access to adequate food and water. Most of the mice that were acclimated to 1% sucrose were determined to exhibit a sucrose preference higher than 70% according to a sucrose preference test (SPT) (Wang et al., 2020). The animals were then randomly assigned to either the control standard group or the CUS group. The mice in the CUS group were housed separately and subjected to stress for 6 weeks. After the depression model was established on day 42, the mice were randomly divided into the CUS/standard group (*n* = 18) or the CUS/GW3965 group (*n* = 21). Beginning in week 7, the mice in the CUS/GW3965 group were intraperitoneally injected with GW3965 at a dose of 10 mg/kg/day (Morales et al., 2008; Cermenati et al., 2010), while those in the CUS/standard and the Control/standard groups were administered the same volume of saline. CUS stimulation was continued during drug treatment. All the procedures conformed to the standards set forth in the National Institutes of Health Guide for the Care and Use of Laboratory Animals, and considerable effort was made to minimize animal suffering.

### Establishment of the CUS model

**FIGURE 1:**
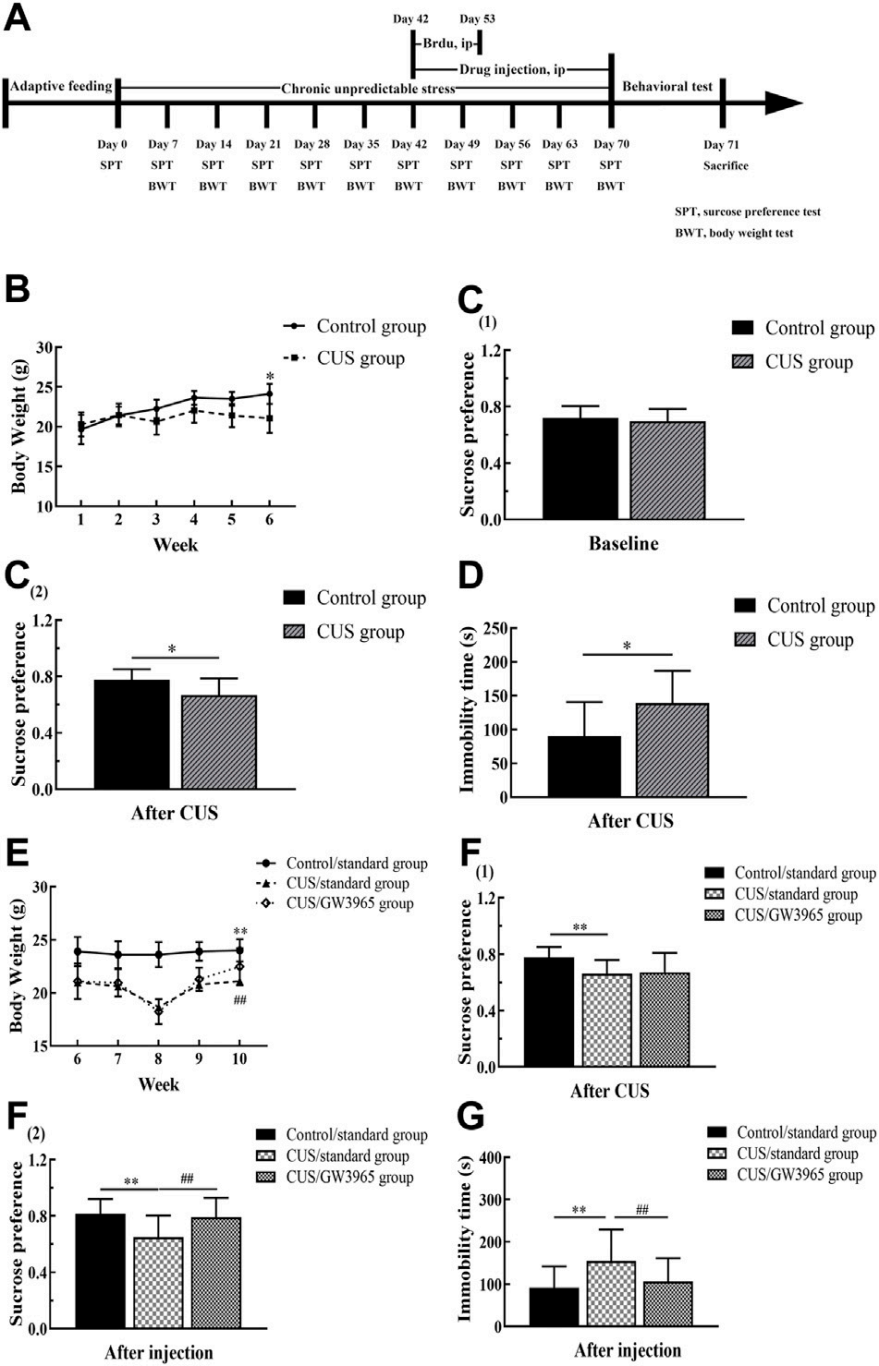
Behavioral experiment timeline. **(A)**The experiment lasted a total of 71 days. The model mice were subjected to a sequence of 10 different stressors (two or three stressors per day for 70 consecutive days). The mice in the CUS/GW3965 group were intraperitoneally injected with GW3965 (10 mg/kg/day) from day 43 to day 70. The mice in the Control/standard group and the CUS/standard group were intraperitoneally injected with the same volume of NS. All the animals were intraperitoneally injected with BrdU (50 mg/kg/day) once a day for 11 consecutive days starting on day 43. Sucrose preference and body weight were assessed on days 7, 14, 21, 28, 35, 42, 49, 56, 63 and 70. SPT: sucrose preference test, BWT, body weight test. **(B)** Changes in the body weights of the mice in the Control group and the CUS group at baseline and during the first 4 weeks of the CUS intervention period. **[C (1)]** The sucrose preference baseline of the mice between the Control group and the CUS group at day 0. **[C (2)]** The sucrose preference difference of the mice between the Control group and the CUS group at day 42. **(D)** Immobility times of the control group and the CUS group in the FST after 6 weeks of CUS intervention. **(E)** Changes in the body weights of the mice among the three groups during GW3965 administration. **[F (1)]** The sucrose preference of the mice among the Control/standard group, CUS/standard and the CUS/GW3965 group at day 42. **[F (2)]** The sucrose preference of the mice among the Control/standard group, CUS/standard and the CUS/GW3965 group at day 70. **(G)** Immobility times of the three groups in the FST after GW3965 administration. ∗ indicates *p <* 0.05 for the CUS group compared with the control group. ∗∗ indicates *p <* 0.05 for the CUS/standard group compared with the Control/standard group. ## indicates *p < 0.05* for the CUS/standard group compared with the CUS/GW3965 group. All the data are shown as the means ± SDs.

The CUS depression model is established by exposing animals to CUS, which causes them to exhibit depressive-like behaviors (anhedonia-like behavior, negative changes in the FST and OFT) (Antoniuk et al., 2019). The procedure was adapted from those described by Willner et al. (Willner et al., 1987) , Banasr et al. (Banasr et al., 2007) and Seney et al. (Seney et al., 2012). The model mice were subjected to a series of interventions, mainly including movement restriction, prohibition of drinking or eating, cold stress, damp bedding, lack of bedding, noise, strobe lights, day and night reversal, cage tilting and table shaking. The mice were subjected to two or three stressors daily throughout the model establishment process, and the same stimulus was not repeated for at least 2 days (Figure 1 and Table 1). Thirty-nine mice in the CUS group exhibited a sucrose preference lower than 65% after being exposed to the stimulations described above lasted for 6 weeks (Liu et al., 2018).

**TABLE 1:** Schedule for the establishment of CUS depression model.

Time	Monday	Tuesday	Wednesday	Thursday	Friday	Saturday	Sunday
Week1	Cage title, Empty bedding, Shaking table	Food deprivation, Restraint	Cold stress, Light off/on	Water deprivation, Electrical foot-shock	Restraint, Exchange bedding	Empty bottle, Noise, Light on	BWT, SPT
Week2	Wet bedding, Cage title, Cold stress	Shaking table, Water deprivation, Electrical foot-shock	Empty bottle, Cold stress, Light on	Shaking table, Noise, Food deprivation	Restraint, Electrical foot-shock	Shaking table, Noise, Exchange bedding	BWT, SPT
Week3	Cage title/Empty bedding, Water deprivation, Electrical foot-shock	Restraint, Empty bottle, Strobe	Cold stress, Noise, Food deprivation	Shaking table, Strobe, Light on	Electrical foot-shock, Cold stress, Water/Food deprivation	Noise, Empty bottle, Shaking table	BWT, SPT
Week4	Shaking table, Exchange bedding, Restraint	Cage title, Empty bedding, Strobe	Restraint, Water deprivation, Noise	Empty bottle, Electrical foot-shock, Light on	Shaking table, Strobe, Cold stress	Electrical foot-shock, Wet bedding	BWT, SPT
Week5	Exchange bedding, Cage title/Empty bedding, Water deprivation	Noise, Restraint, Food deprivation	Empty bottle, Cold stress, Water deprivation	Shaking table, Restraint, Light off/on	Electrical foot-shock, Strobe, Cold stress	Empty bottle, Light on	BWT, SPT
Week6	Cage title, Empty bedding, Shaking table	Food deprivation, Restraint	Cold stress, Light off/on	Water deprivation, Electrical foot-shock	Restraint, Exchange bedding	Empty bottle, Noise, Light on	BWT, SPT FST
Week7	Wet bedding, Cage title, Cold stress	Shaking table, Water deprivation, Electrical foot-shock	Empty bottle, Cold stress, Light on	Shaking table, Noise, Food deprivation	Restraint, Electrical foot-shock	Shaking table, Noise, Exchange bedding	BWT, SPT
Week8	Cage title/Empty bedding, Water deprivation, Electrical foot-shock	Restraint, Empty bottle, Strobe	Cold stress, Noise, Food deprivation	Shaking table, Strobe, Light on	Electrical foot-shock, Cold stress, Water/Food deprivation	Noise, Empty bottle, Shaking table	BWT, SPT
Week9	Shaking table, Exchange bedding, Restraint	Cage title, Empty bedding, Strobe	Restraint, Water deprivation, Noise	Empty bottle, Electrical foot-shock, Light on	Shaking table, Strobe, Cold stress	Electrical foot-shock, Wet bedding	BWT, SPT
Week10	Exchange bedding, Cage title/Empty bedding, Water deprivation	Noise, Restraint, Food deprivation	Empty bottle, Cold stress, Water deprivation	Shaking table, Restraint, Light off/on	Electrical foot-shock, Strobe, Cold stress	Empty bottle, Light on	BWT, SPT FST OFT

### Drug and bromodeoxyuridine (BrdU) injection

In the CUS/GW3965 group, the animals were intraperitoneally injected with the LXR agonist GW3965 (Selleckchem, S2630, 10 mg/kg/day) for 28 consecutive days (Collins et al., 2002; Morales et al., 2008). The Control/standard group and the CUS/standard group were intraperitoneally injected with the same volume of normal saline (Figure 1A). During the periods of administration, the mice in the CUS/standard group and the CUS/GW3965 group were subjected to the stressors. In the present experiment, BrdU was diluted to a concentration of 10 mg/ml in normal saline. All the animals were intraperitoneally injected with BrdU at a dosage of 50 mg/kg/day for 11 consecutive days starting in the sixth week. The concentration and dosage adopted to evaluate the effect of GW3965 on cell proliferation were referred to the previous studies (Wojtowicz and Kee, 2006; HG and CM, 2007; Qiu et al., 2007; Mateus-Pinheiro et al., 2013; Tang et al., 2021; Liang et al., 2022).

### Body weight assessment

Changes in body weight can reflect the growth and health of animals. At the beginning of the experiment, the body weights of the mice were recorded regularly every week.

### Behavioral tests

#### SPT

The SPT is a classical behavioral test used to evaluate rodent anhedonia (Willner et al., 1987; Banasr et al., 2007). Prior to the first test, all the mice underwent sucrose solution (1%) adaptation, which included drinking from of two bottles of sucrose solution (1%) for 24 h and then drinking from one bottle of water and one bottle of sucrose solution (1%) for 24 h. In the formal experiment, each mouse was given a bottle full of water and a bottle full of sucrose solution (1%), which were randomly placed on the left or right side of the compartment. After 24 h, the sucrose consumption and water consumption were determined. The sucrose preference percentage was calculated as the percentage of sucrose intake relative to the total fluid intake (water + sucrose). The SPT was performed at every weekend.

#### FST

The FST is a behavioral test widely used to assess the effects of antidepressants (Petit-Demouliere, Chenu and Bourin, 2005). During the formal test, each mouse was gently placed in a transparent resin bucket (60 cm in height, 10 cm in diameter) filled with water (45 cm depth, temperature 23–25°C) for 6 min. A camera recorded a video of each mouse swimming, and the immobility time in the last 5 min was quantified. After each mouse was tested, the water in the bucket was changed.

#### OFT

The OFT is a method used to evaluate autonomous behavior and exploratory behavior in novel environments (Huang et al., 2017), which reflects anxiety-like behavior. The OFT apparatus included a white acrylic box and a camera. Each mouse was placed in the center of box (50 × 50 × 45 cm). The camera recorded the distance each mouse traveled in the central area, the time spent traveling in the central area and the number of entries into the central area over 6 min. In addition, the activity in the last 5 min was quantified. At the end of each test, the box was cleaned with 70% ethanol.

### Perfusion and tissue preparation

Five mice were randomly selected from each group and anesthetized with 1% pentobarbital sodium, which was administered intraperitoneally at a dose of 40 ml/kg. The mice were then perfused with 4% paraformaldehyde in 0.6 M phosphate-buffered saline (pH 7.4). One brain hemisphere was randomly selected for continuous sectioning, and each fifth of the tissue was cut into equal (50-μm-thick) sections. Each group of sections was placed in a test tube containing 0.01 M PBS. Every set of serial sections was washed with 0.01 M PBS (pH 7.4) and 75% alcohol three times. A total of five groups of sections were generated from each brain hemisphere, and the sections were stored at −20°C.

### Immunohistochemistry and stereological analysis

**FIGURE 2:**
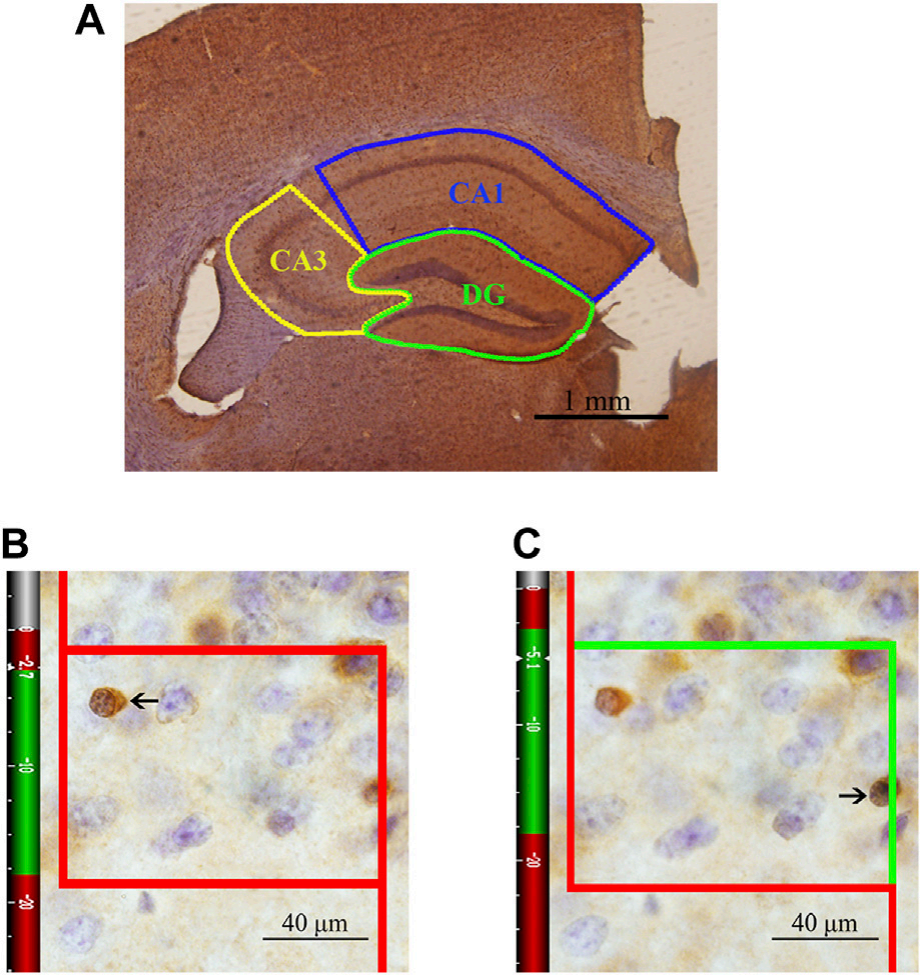
Schematic diagram of the subregions of the hippocampus and an illustration of the optical fractionator technique for counting of CC1^+^ cells. **(A)** The area indicated by the solid blue line represents the CA1 area, the area indicated by the solid yellow line represents the CA3 area, and the area indicated by the solid green line represents the DG. Bar = 1 mm. **(B)** The CC1^+^ cells that are clearly in focus in the guard zone were not counted. **(C)** The CC1^+^ cells that are clearly in focus in the counting zone, as indicated by the arrows, were counted. Bar = 40 μm.

One set of sections was randomly selected from each animal and washed with 0.01 M PBS (pH 7.4, six times, 10 min each) and then thoroughly washed with 0.01 M PBS supplemented with 0.3% Triton X-100 and 0.1% Tween-20 (PBS + T) (six times, 10 min each). The sections were sequentially incubated with 3% hydrogen peroxide for 20 min and incubated in a 0.01 M citrate buffer solution (pH 6.0) in a water bath for 15 min for antigen retrieval. Then, the sections were blocked with working buffer consisting of 10% normal goat serum (SP 9002-A) and 1% fetal bovine serum in PBS + T for 2 h at 37°C and sequentially incubated with the working buffer and a mouse anti-APC primary antibody (marking CC1, Millipore, OP80, 1:1000) diluted in PBS + T for 48 h at 4°C. After washing in PBS + T (six times, 10 min each), the sections were incubated with biotinylated goat anti-mouse immunoglobulin (SP 9002-B, 1:10) diluted in PBS + T for 3 h at 37°C. After washing in PBS + T (six times, 10 min each), the sections were incubated in horseradish peroxidase-conjugated streptavidin (SP 9002-C) for 2 h at 37°C. Then, the sections were repeatedly rinsed in PBS, transferred to diaminobenzidine (DAB) and incubated for approximately 1 min, dehydrated in a graded ethanol series (75, 85, 95, 100, and 100%, 3 min each), cleared using xylene (three times, 10 min each) and mounted. After immunohistochemical staining, the CA1, CA3 and DG regions were accurately traced and analyzed under a light microscope at ×4 magnification with a stereological system (ZEISS, Germany) (Figure 2). The numbers of CC1^+^ cells in the three regions of the hippocampus were counted using the optical fractionator method in each section under an oil objective lens at ×100 magnification (N.A. 1.40). Optical fractionator counting was performed in the delineated regions of the sections in a systematic, random fashion so that each section had an equal probability of being sampled and the intervals between sections and counting sites were constant. The total number of OLs (N) was estimated according to the following equation (West, Slomianka and Gundersen, 1991):

N = ΣQ− × (1/ssf) × (1/asf) ×(1/hsf) 

where ΣQ^−^ is the number of CC1^+^ cells actually counted in the specimens, ssf is the section sampling fraction, asf is the area sampling fraction, and hsf is the thickness sampling fraction. In this study, every fifth section from a random starting point in the series was sampled for analysis so that the ssf was 1/5. Subsequently, the ratio between the area of the unbiased counting frame and the rectangular area that was obtained by multiplying the step length in the *x*-axis and the step length in the *y*-axis was considered the asf, which was 8% in the DG region and 6% in the CA1/CA3 region. The mean thickness of each section (t) was approximately 30 μm. A “guard zone” of 3 μm was measured from the surface of each section, and the CC1^+^ cells were counted to a depth of 15 μm below the “guard zone” (h, height of the dissector). The last fraction, hsf, was therefore calculated as h/t = 15/30. An unbiased counting frame is shown in Figures 2B,C. The solid line of the frame and its extension show the exclusion lines, and the dotted line of the frame shows the inclusion line. When the inclusion (dotted) line was green, the CC1^+^ cells that were completely inside the counting frame or partly inside the counting frame but only touching the inclusion (dotted) line were counted.

### Immunofluorescence analysis

After being washed with 0.01 M PBS and PBS+T, free-floating sections were incubated in blocking buffer containing 1% fetal bovine serum, 5% normal goat serum (SP 9002-A), and PBS + T for 2 h at 37°C. Then, the sections were stained with a mouse anti-APC primary antibody and a rabbit anti-Olig2 primary antibody (Abcam, ab109186, 1:1000) for 48 h at 4°C. The sections were then incubated at 37°C for 2 h with DyLight 549-conjugated goat anti-mouse IgG and DyLight 488-conjugated goat anti-rabbit IgG in the dark and then washed in the dark with PBS for 30 min on the third day. The sections were stained with a rat anti-BrdU primary antibody (Abcam, ab6326, 1:500) for 48 h at 4°C (after treatment with 2 mol/l HCl for 50 min at 37°C and boric acid for 20 min at room temperature).

After the sections were washed with PBS + T for 30 min on the sixth day, they were incubated at 37°C for 2 h with DyLight 405-conjugated goat anti-rat IgG. Then, the sections were mounted on gelatin-coated slides with antifade solution to reduce fluorescence quenching. Complete images of the hippocampus were obtained using laser scanning confocal microscopy (Nikon, Japan), and cells were counted after random and equidistant sampling of each subregion (NIS-Elements, 4.5). The numbers of Olig2^+^/BrdU^+^ cells and CC1^+^/Olig2^+^/BrdU^+^ cells were estimated. After being washed, free-floating sections were incubated in blocking buffer containing 1% fetal bovine serum and 5% SP 9001-A and then stained with a rabbit anti-MBP primary antibody for 48 h at 4°C. The sections were incubated with DyLight 488-conjugated goat anti-rabbit IgG in the dark and then washed in the dark with PBS on the third day. A complete image of each immunofluorescent section was compared to a reference section stained with DAPI to locate the subregions of the hippocampus. MBP immunostaining intensity was quantified using Image-Pro Plus Software. The same laser intensity was used in the MBP fluorescence intensity experiment.

### Statistical analyses

All the data collected were analyzed with SPSS (ver. 19.0, SPSS Inc., Chicago, United States). All the data, except the MBP density data, were expressed as the mean ± standard deviation (SD). Levene’s test was used to evaluate the similarities of variances among the groups. All the data, except the MBP density data, were normally distributed. The effect of CUS on body weight was analyzed using repeated-measures ANOVA. If the sphericity assumption was met, Mauchly’s test was used; otherwise, the Greenhouse–Geisser test was used. Student’s t-test was used to compare the other results between two groups (the Control group and CUS group). One-way ANOVA was used to compare the results among three groups (the Control/Standard group, the CUS/standard group and the CUS/GW3965 group). If the data displayed similar variance among groups, a least significant difference (LSD) *post hoc* test was used for analyses; otherwise, Tamhane’s T2(M) post hoc test was used for analyses. Nonparametric tests were used to analyze the MBP density data, which did not conform to a normal distribution. The MBP density data were expressed as upper and lower quartiles. *p* < 0.05 was considered to indicate statistical significance in all tests.

## Results

### GW3965 alleviated depressive the behaviors induced by CUS

During the CUS intervention period, the mice exposed to CUS lost weight, while the control animals maintained stable weights. At the end of the sixth week, the body weight of the CUS group was significantly lower than that of the Control group (*p* < 0.001, Figure 1B). After 4 weeks of GW3965 or normal saline injection, the body weights of the mice in the CUS/standard group were significantly lower than those in the Control/standard group, and those in the CUS/GW3965 group were significantly higher than those in the CUS/standard group [F (2,45) = 7.723, *p* = 0.001; *p*< 0.001 for Control/standard vs CUS/standard; *p* = 0.036 for CUS/standard vs CUS/GW3965, Figure 1E].

In the SPT, there was no significantly difference in sucrose preference between the mice in the Control group and the CUS group before the CUS intervention [*p* > 0.05, Figure 1C (1)]. After 6 weeks of CUS intervention, the mice in the CUS group showed a significantly lower sucrose preference than the mice in the Control group [*p* = 0.006, Figure 1C (2)]. Before the starting of the drug injection, the mice in the CUS group were randomly divided into the CUS/standard group and the CUS/GW3965 group so that there was no significantly difference in sucrose preference between the mice in the CUS/standard group and the CUS/GW3965 group [*p* > 0.05, Figure 1F (1)]. At the end of the drug injection period, the sucrose preference percentage in the CUS/standard group was significantly lower than that in the Control/standard group [F (2,45) = 6.548, *p* = 0.003; *p* = 0.006 for Control/standard vs CUS/standard, Figure 1F (2)]. In addition, the sucrose preference percentage in the CUS/standard group was significantly lower than that in the CUS/GW3965 group [*p* = 0.003 for CUS/standard vs CUS/GW3965, Figure 1F (2)].

In the FST, after 6 weeks of CUS intervention, the mice in the CUS/standard group had a significantly longer immobility time than the mice in the Control/standard group (*p* = 0.016, Figure 1D). At the end of the drug injection period, the immobility time in the CUS/standard group was significantly longer than that in the Control/standard group [F (2,45) = 4.273, *p* = 0.020; *p* = 0.017 for Control/standard vs CUS/standard, Figure 1G). In addition, the immobility time in the CUS/standard group was significantly longer than that in the CUS/GW3965 group (*p* = 0.019 for CUS/standard vs CUS/GW3965, Figure 1G).

In the OFT, the time spent traveling in the central area was significantly shorter and the number of entries into the central area was significantly lower in the CUS/standard group than in the Control/standard group, while there were no significant differences in these variables between the CUS/standard group and the CUS/GW3965 group [the time spent traveling: F (2,45) = 5.396, *p* = 0.008; *p* = 0.002 for Control/standard vs CUS/standard; *p* = 0.453 for CUS/standard vs CUS/GW3965; the number of entries: F (2,45) = 5.396, *p* = 0.007, *p* = 0.004 for Control/standard vs CUS/standard; *p* = 0.978 for CUS/standard vs CUS/GW3965, Table 2]. In addition, we found that the traveled distance in the central area was similar among the Control/standard group, CUS/standard group and CUS/GW3965 group [F (2,45) = 2.399, *p* = 0.108, *p* = 0.073 for Control/standard vs CUS/standard; *p* = 0.795 for CUS/standard vs CUS/GW3965, Table 2].

**TABLE 2:** Comparisons of the OFT results among the Control/standard group, the CUS/standard group and the CUS/standard GW3965 group after GW3965 intervention.

	Control/standard group	CUS/standard group	CUS/GW3965 group
Traveled time in the central area (s)	54.38 ± 37.14	20.22 ± 12.04^a^	26.19 ± 23.91
Traveled distance in the central area (m)	4.19 ± 1.47	2.69 ± 1.17^a^	2.58 ± 1.11
Number of entries into the central area	22.22 ± 7.69	12.22 ± 4.65^a^	12.33 ± 4.97

a Indicates *p <* 0.05 for the CUS/standard group compared with the Control/standard group. All the data are shown as the means ± SDs.

These results indicated that chronic stress induced depressive-like behavior and anxiety-like behavior and that GW3965 significantly ameliorated depressive-like behavior but did not affect anxiety-like behavior.

### GW3965 increased the numbers of mature OLs in the hippocampal subregions of CUS-exposed mice

**FIGURE 3:**
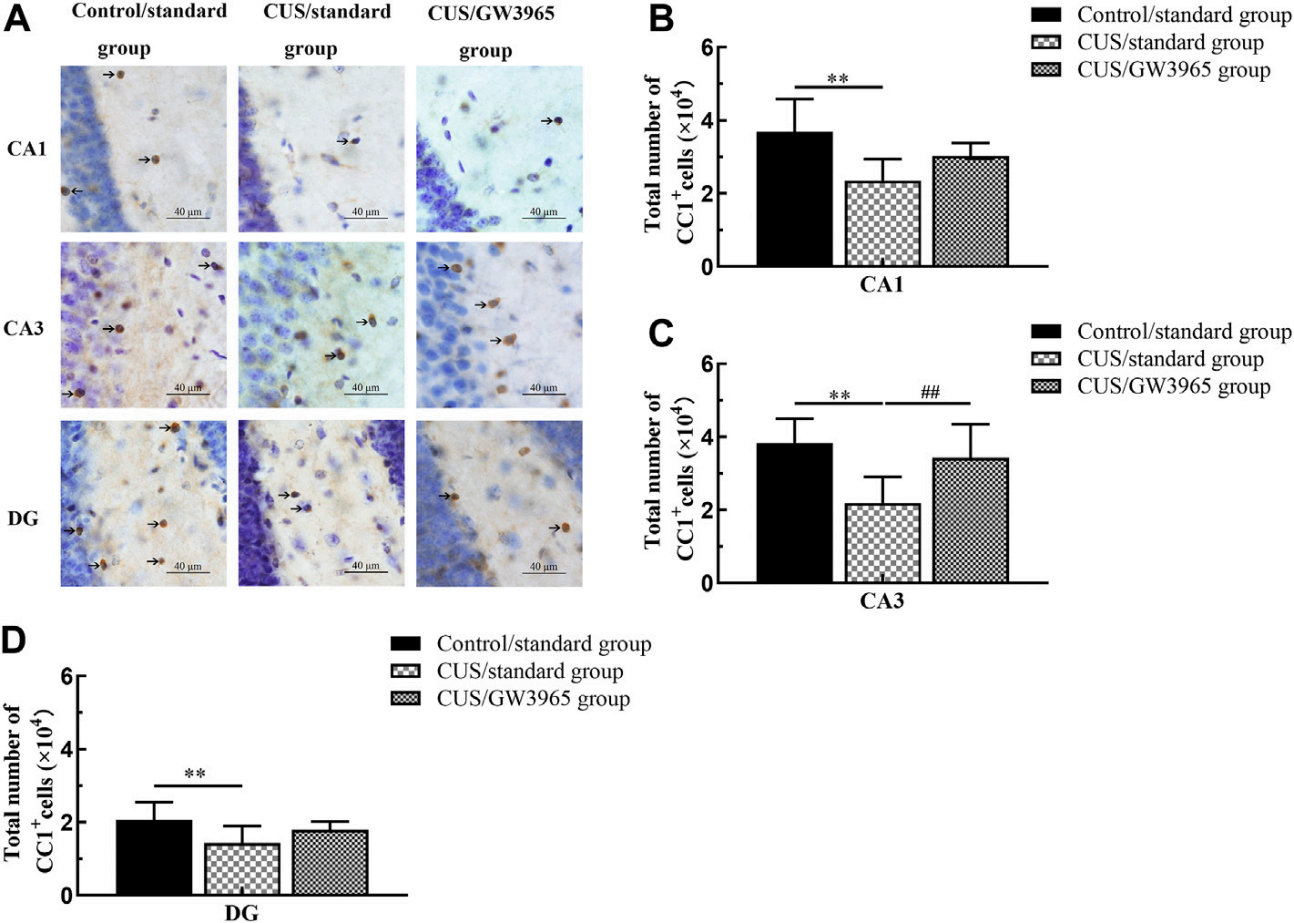
Illustration of CC1^+^ cell density. **(A)** Representative images of immunohistochemical staining of CC1^+^ cells in the CA1, CA3 and DG areas of the hippocampus in the Control/standard group, the CUS/standard group and the CUS/GW3965 group. The arrows indicate CC1^+^ cells. Bar = 40 μm. **(B)** Comparisons of the CC1^+^ cell numbers in CA1 region among the Control/standard group, the CUS/standard group and the CUS/GW3965 group. **(C)** Comparisons of the CC1^+^ cell numbers in CA3 region among the Control/standard group, the CUS/standard group and the CUS/GW3965 group. **(D)** Comparisons of the CC1^+^ cell numbers in DG region among the Control/standard group, the CUS/standard group and the CUS/GW3965 group. ∗∗ indicates *p <* 0.05 for the CUS/standard group compared with the Control/standard group. ## indicates *p <* 0.05 for the CUS/standard group compared with the CUS/GW3965 group. All the data are shown as the means ± SDs.

Representative images of immunohistochemical staining with an anti-APC antibody (CC1^+^ cell marker) are shown in Figure 3A. The mean total numbers of CC1^+^ cells in the three hippocampal subregions of the three groups are shown in Figure 3B. These results indicated that the numbers of CC1^+^ cells in the CA1, CA3 and DG regions were significantly lower in the CUS/standard group than in the Control/standard group [CA1: F (2,12) = 5.260, *p* = 0.023; *p* = 0.007 for Control/standard vs CUS/standard, Figure 3B; CA3: F (2,12) = 6.086, *p* = 0.015, *p* = 0.006 for Control/standard vs CUS/standard , Figure 3C; DG: F (2,12) = 3.042, *p* = 0.085; *p* = 0.030 for Control/standard vs CUS/standard, Figure 3D] and that the number of CC1^+^ cells in the CA3 region was significantly higher in the CUS/GW3965 group than in the CUS/standard group [CA3: F (2,12) = 6.086, *p* = 0.015; *p* = 0.026 for CUS/standard vs CUS/GW3965, Figure 3C]. However, the numbers of CC1^+^ cells in the CA1 and DG regions did not differ between the CUS/standard group and the CUS/GW3965 group [CA1: F (2,12) = 5.260, *p* = 0.023; *p* = 0.126 for CUS/standard vs CUS/GW3965, Figure 3B; DG: F (2,12) = 3.042, *p* = 0.085; *p* = 0.178 for CUS/standard vs CUS/GW3965, Figure 3D]. These results indicated that GW3965 treatment significantly reversed the chronic stress-induced decreases in the numbers of OLs.

### GW3965 increased the density of newborn and mature OLs in the hippocampal subregions of CUS-exposed mice

**FIGURE 4:**
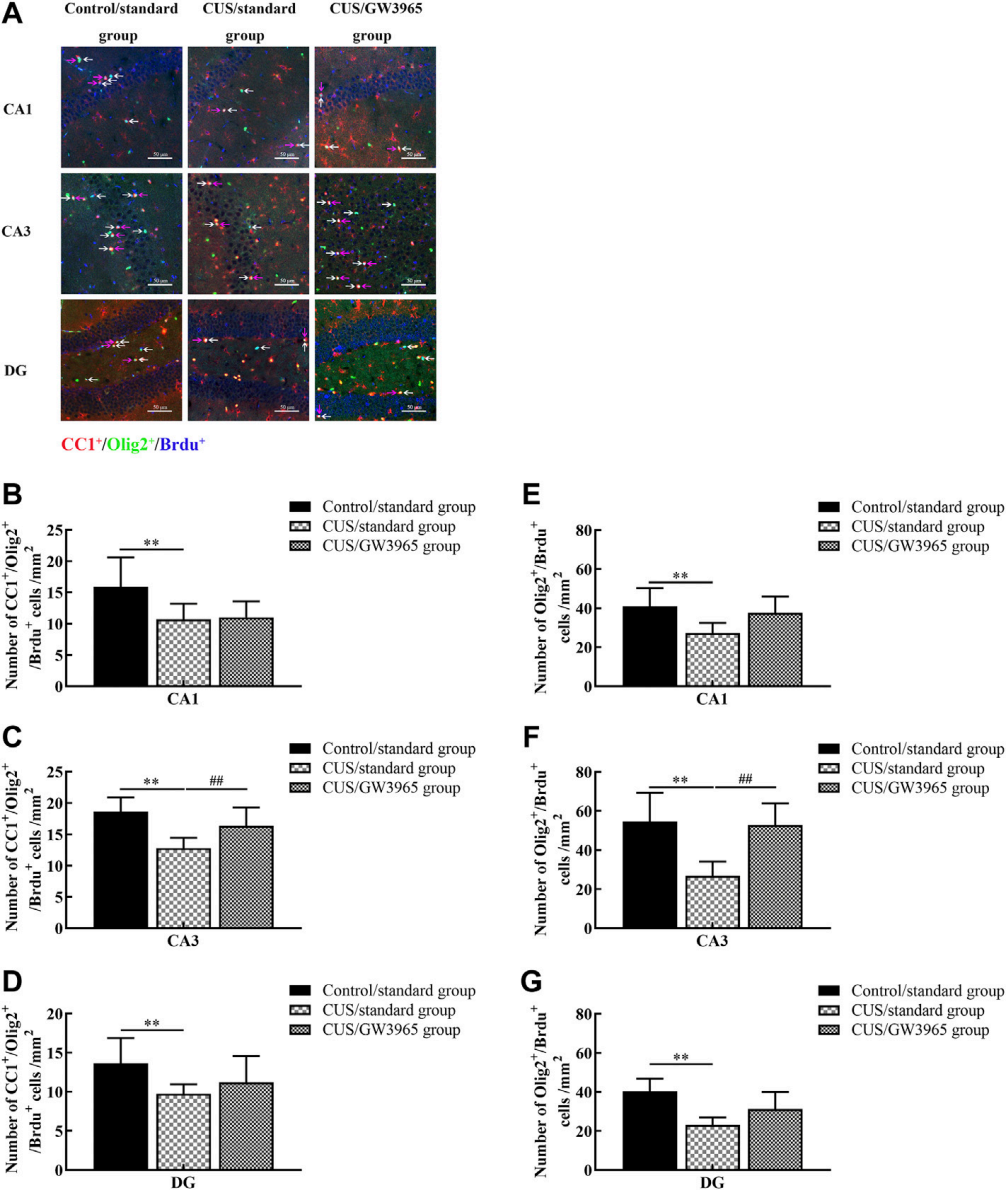
Illustration of the Olig2^+^/BrdU^+^ and CC1^+^/Olig2^+^/BrdU^+^ cell densities. **(A)** Representative images of the immunofluorescence staining of Olig2^+^/BrdU^+^ cells and CC1^+^/Olig2^+^/BrdU^+^ cells in the CA1, CA3 and DG areas of the hippocampus in the Control/standard group, the CUS/standard group and the CUS/GW3965 group. CC1^+^: red, Olig2^+^: green, BrdU^+^: blue. The white arrows indicate Olig2^+^/BrdU^+^ cells. The red arrows indicate CC1^+^/Olig2^+^/BrdU^+^ cells. Bar = 50 μm. **(B)** Comparisons of the CC1^+^/Olig2^+^/BrdU^+^ cell densities in CA1 region among the three groups. **(C)** Comparisons of the CC1^+^/Olig2^+^/BrdU^+^ cell densities in CA3 region among the three groups. **(D)** Comparisons of the CC1^+^/Olig2^+^/BrdU^+^ cell densities in DG region among the three groups. **(E)** Comparisons of the Olig2^+^/BrdU^+^ cell densities in CA1 region of the hippocampus among the three groups. **(F)** Comparisons of the Olig2^+^/BrdU^+^ cell densities in CA3 region among the three groups. **(G)** Comparisons of the Olig2^+^/BrdU^+^ cell densities in DG region among the three groups. ∗∗ indicates *p <* 0.05 for the CUS/standard group compared with the Control/standard group. ## indicates *p <* 0.05 for the CUS/standard group compared with the CUS/GW3965 group. All the data are shown as the means ± SDs.

We hypothesized that the increases in the numbers of hippocampal OLs in the mice occurred due to changes in the numbers of newborn OLs. To test this hypothesis, we quantified the numbers of cells that were positive for Olig2 (a marker of the OL lineage), CC1 (a marker of mature OLs) and BrdU (a marker of newborn cells) expression according to immunofluorescence (Figure 4A).

We quantified the number of CC1^+^/Olig2^+^/BrdU^+^ cells in the three subregions (CA1, CA3 and DG) of the hippocampus and found that the densities of CC1^+^/Olig2^+^/BrdU^+^ cells in the CUS/standard group were significantly lower than those in the Control/standard group [CA1: F (2,12) = 3.617, *p* = 0.059; *p* = 0.034 for Control/standard vs CUS/standard, Figure 4B; CA3: F (2,12) = 7.826, *p* = 0.007; *p* = 0.002 for Control/standard vs CUS/standard, Figure 4C; DG: F (2,12) = 2.467, *p* = 0.127; *p* = 0.048 for Control/standard vs CUS/standard, Figure 4D]. Similar results were observed regarding the densities of Olig2^+^/BrdU^+^ cells in the CA1, CA3 and DG regions [CA1: F (2,12) = 4.107, *p* = 0.044; *p* = 0.018 for Control/standard vs CUS/standard, Figure 4E; CA3: F (2,12) = 9.341, *p* = 0.004; *p* = 0.002 for Control/standard vs CUS/standard, Figure 4F; DG: F (2,12) = 8.376, *p* = 0.005; *p* = 0.001 for Control/standard vs CUS/standard, Figure 4G]. Moreover, the density of CC1^+^/Olig2^+^/BrdU^+^ cells in the CA3 region in the CUS/GW3965 group was especially significantly higher than that in the CUS/standard group [F (2,12) = 7.826, *p* = 0.007; *p* = 0.034 for CUS/standard vs CUS/GW3965, Figure 4C]. A similar result was observed regarding the density of Olig2^+^/BrdU^+^ cells in the CA3 region [F (2,12) = 9.341, *p* = 0.004; *p* = 0.004 for CUS/standard vs CUS/GW3965, Figure 4F]. These results indicated that GW3965 had a beneficial effect on the generation of newborn OLs in the CA3 region mice exposed to stress.

### GW3965 increased the intensity of MBP staining in the CA3 subregion of CUS-exposed mice

**FIGURE 5:**
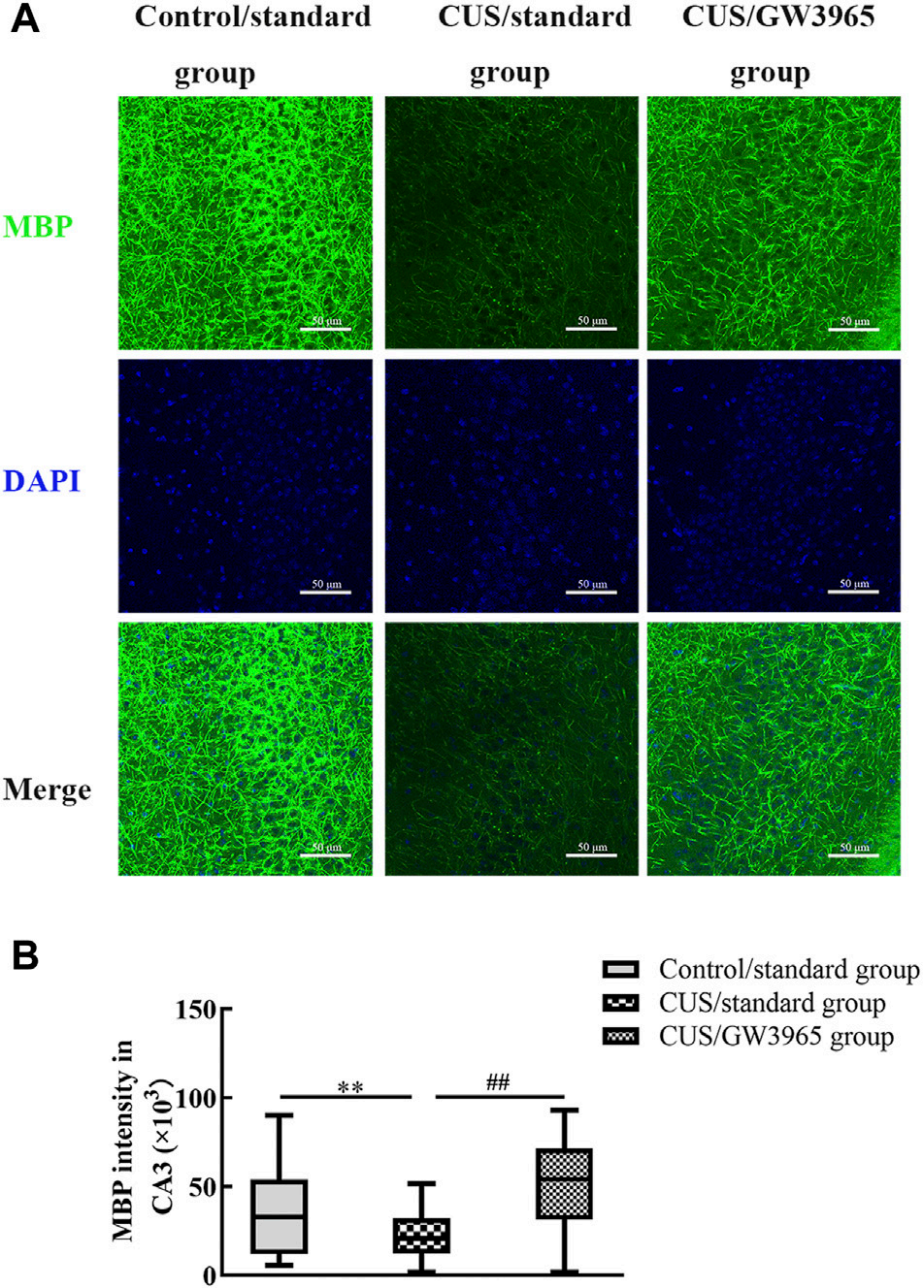
Illustration of the MBP staining intensity. **(A)** Representative images of immunofluorescence staining of MBP in the CA3 subregion in the three groups. MBP: green, DAPI: blue. Bar = 50 μm. **(B)** Comparisons of the MBP staining intensities in different subregions of the hippocampus among the three groups. ∗∗ indicates *p <* 0.05 for the CUS/standard group compared with the Control/standard group. ## indicates *p <* 0.05 for the CUS/standard group compared with the CUS/GW3965 group. All the data are shown as the upper and lower quartiles.

To assess the effect of the LXR agonist on the myelin sheath, immunofluorescence was used to assess the expression of MBP in the CA3 region in each group of mice (Figure 5A). The MBP intensity in the CA3 region was significantly lower in the CUS/standard group than in the Control/standard group (*p* = 0.001, Figure 5B) and significantly higher in the CUS/GW3965 group than in the CUS/standard group. These results indicated that GW3965 improved myelination in the CA3 region in CUS mice (*p* < 0.001, Figure 5B).

## Discussion

Chronic stress is recognized as a major risk factor for depression, and the CUS model is the animal model that is most commonly used in studies on depression (Hu et al., 2017; Khan et al., 2020). This model can simulate various stress events encountered by humans. In the present study, a series of behavioral tests were conducted to assess the depressive-like and anxiety-like behaviors of mice exposed to CUS and to evaluate the antidepressant effects of an LXR agonist. We observed that the body weights and sucrose preferences of CUS mice were significantly lower than those of control mice. In contrast, the immobility time in the FST was obviously higher for CUS mice than for control mice. Both the time spent and the distance traveled in the inner area as well as the number of entries into the central area were significantly higher in the CUS/standard group than in the control group in the OFT. These results indicated that the depression model was successfully established in this study. After 4 weeks of LXR agonist treatment, body weight changes and behavioral changes observed in the SPT and FST were markedly attenuated in the CUS/GW3965 group, while the behavioral changes observed in the OFT were not. These results confirmed that LXR agonist treatment ameliorated anhedonia-like and despair-like behaviors except the anxiety-like behaviors in the model mice. These results are consistent with those of Peng et al.’s previous study (Peng et al., 2018), and Xu et al. confirmed that GW3965 prevented CUMS-induced depression- and anxiety-like behaviors (Xu et al., 2021). Our experiment was slightly different from that of Peng et al. and Xu et al. In the present study, GW3965 was intraperitoneally injected into mice at a dosage of 10 mg/kg/day for 28 consecutive days, while Peng et al. treated animals with 5 mg/kg/day for 26 consecutive days, and Xu et al. injected mice with GW3965 at a dosage of 20 mg/kg/day for 26 consecutive days. Notably, there is reliable evidence indicating that the LXR agonist GW3965 works well at a dose of 10 mg/kg (Morales et al., 2008; Cermenati et al., 2010). It’s obvious that the different dosages of GW3965 may result in the inconsistencies in depressive phenotypes. On the other hand, we began to administer GW3965 after 6 weeks of CUS intervention to explore the antidepressant effect of the LXR agonist. However, Peng et al. began drug treatment in the middle of the CUS intervention and Xu et al. began drug treatment in the beginning of CUMS intervention; thus, their findings may reflect the preventative effects of the LXR agonist on pathogenesis to a greater extent than our findings (Peng et al., 2018; Xu et al., 2021). Our results indicated that the LXR agonist improved CUS-induced depressive-like behaviors, and our results might provide an important behavioral basis for further exploration of the mechanism by which this compound functions in the treatment of depression.

An increasing number of studies have found that changes in mature OLs are important pathological changes in depression and have indicated that promoting OL proliferation and differentiation may be a potential mechanism for the reversal of depressive-like behaviors (Hagemeyer et al., 2012; Liu et al., 2012). These findings have led to a glial-inclusive viewpoint in which glial cells are believed to contribute to the pathophysiology of depression. Previous studies have shown significant decreases in OL density in the pregenual anterior cingulate cortex (pACC)-adjacent white matter of MDD patients (Mosebach et al., 2013) and decreases in cell proliferation in the cerebral cortices of adult rats subjected to CUS (Banasr et al., 2007). Birey et al. also found that the density of NG2^+^ glial cells in the hippocampus appears to be significantly reduced in mice exposed to chronic social defeat (Birey et al., 2015). In the current study, we used immunohistochemistry combined with a stereological method to quantify the numbers of CC1^+^ cells and found decreased numbers of mature OLs in the CA1 region, CA3 region and DG of the hippocampus in the CUS + NS group. Furthermore, using immunofluorescence, we found that the density of Olig2^+^/BrdU^+^ cells and the density of CC1^+^/Olig2^+^/BrdU^+^ cells were similarly decreased. These studies further confirmed that the numbers of proliferating or differentiating OLs in the hippocampus were reduced in the context of depression. As recent studies have indicated, the efficacy of many currently available clinical therapeutics may be due to treatment-induced changes in glia, particularly OLs, and their myelin (Bartzokis, 2012). Studies have shown that electroconvulsive therapy and running exercise can benefit OL proliferation or OL maturation in the hippocampus, which might form an important structural basis for antidepressant effects (Bromis et al., 2018; Luo et al., 2019; Tang et al., 2019, 2021).

These findings suggest that the maturation of OLs may be one of the key mechanisms underlying the efficacy of antidepressants treatment. Consistent with this idea, previous studies have demonstrated that LXRβ (−/−) mice exhibited reduced OPC production from RGCs along the germinal ventricular zone-subventricular zone and corpus callosum and an LXR agonist led to increased differentiation cultured RGCs into OPCs (Xu et al., 2014). We hypothesized that favorable effects on OLs might form an important cellular basis for the LXR agonist-mediated amelioration of despair-like behaviors. In our next experiment, we found that the LXR agonist GW3965 specifically increased the numbers of CC1^+^ cells and the densities of Olig2^+^/BrdU^+^ cells and CC1^+^/Olig2^+^/BrdU^+^ cells in the CA3 region but not in the CA1 or DG region, confirming that the LXR agonist might have a protective effect on OLs in the CA3 in CUS model mice. Thus, the data in the present study indicated that OLs in the CA3 region acted as unique structural targets for the antidepressant effects of the LXR agonist. Previous studies found that the impaired oligodendrocyte maturation and myelination in hippocampus were relative to the depressive behaviors (Bromis et al., 2018; Luo et al., 2019; Tang et al., 2019, 2021). Compared with CA1 and DG, the apical branch and the dendritic length in the pyramidal neurons of CA3 region were changed when exposed to stress (Wang et al., 2021). The density of MBP immunoreactive fibers in the CA3 stratum pyramidal was generally highest in the adult mice compared to CA1 and DG (Ahn et al., 2017). Therefore, it is generally supposed that the oligodendrocytes which are forming the myelin sheath and wrapping axons of pyramidal neurons are more vulnerable to stress in the CA3 compared to the CA1 and DG (Mastrodonato et al., 2018; Krzystyniak et al., 2019; Tian et al., 2019). The oligodendrocyte and myelin damage and degeneration were found in the CA3 of the hippocampus in schizophrenia patients (Uranova et al., 2017). The above studies suggested that the pathological changes of myelin sheaths in CA3 may be closely related to dysthymic disorders. If this change is reversed, it is likely to improve those disorders. In the current study, GW3965 promoted OPC differentiation only in the CA3 subfield, thereby potentially rescuing the deficits in this region of the hippocampus and subsequent depressive-like behaviors. Liu, Q et al. found that stereotactic injection of NEP1-40 into the CA3 promoted oligodendrocyte progenitor cell differentiation in mice exposed to the CPZ intervention (Liu et al., 2020). The previous findings may partly support our findings that LXR agonist had a protective effect on OLs only in the CA3 region. However, the exact reason for this remains to be answered. Moreover, whether there is a causal link between the GW3965-induced proliferating OPCs in the CA3 subfield and GW3965-induced improvement of depressive-like behaviors remains to be answered.

Myelination is one of the major contributors to expanding neural plasticity in the human brain and is essential for neuronal development and function (Meffre et al., 2015; Sacchet and Gotlib, 2017). Emerging evidence has shown that myelination-related transcriptional genes that are important for myelin structure (CNP, MAG, MOG) are significantly downregulated in patients with major depressive disorder (Aston, Jiang and Sokolov, 2005). Preclinical studies reported abnormal myelination in other depression models, such as mice subjected to social isolation (Liu, et al., 2012; Makinodan et al., 2017), olfactory bulbectomized (OBX) mice (Takahashi et al., 2021) and mice subjected to chronic social defeat stress (CSCD) (Lehmann et al., 2017). Takahashi et al. reported that OBX mice exhibited impaired myelination along with impaired mature oligodendrocytes in the PFC, which were relevant to depressive-like behavior (Takahashi et al., 2022). Makinodan et al. showed that mice isolated for 2 weeks exhibited alterations in myelination in the prefrontal cortex (Makinodan et al., 2012). Liu et al. found that clemastine could promote adult myelination and OL differentiation in the mPFC, which suggested a potential strategy for reversing depressive-like social behavior (Liu et al., 2016). However, the relationship between GW3965 and myelination is still unclear. To further confirm the effects of GW3965 on myelination, we used immunofluorescence to estimate the expression of MBP in the CA3 in order to explore the effect of the LXR agonist on myelination. Consistent with the observations described above, the MBP staining intensity in the CUS/GW3965 group was significantly higher than that in the CUS/standard group. Therefore, we hypothesized that the repair of abnormal OLs caused by GW3965 might contribute to the recovery of myelination in the CA3 region. LXRs could regulate the myelination and remyelination processes by mediating OLs, which provide the myelin sheath (Nugent et al., 2019; Berghoff et al., 2021). Consistent with this mechanism, Meffre D et al. found that the LXR agonist TO901317 increases the expression of MBP and PLP after demyelination in the cerebellum (Meffre et al., 2015). While LXRs are known to play prominent regulatory roles in cerebellar myelination, Xu et al. showed that LXRβ is also critical for corpus callosum myelination (Xu et al., 2014). Taken together, the evidence suggests that regulation of myelination by LXRs plays an important role in the pathology of depression. However, the mechanisms by which LXR agonists regulate OL differentiation, proliferation and myelination in the CA3 region of the hippocampus remain to be further studied.

In the present study, we used CUS to establish a mouse model of depression and proved that an LXR agonist alleviated depressive-like behaviors, attenuated OL abnormalities and improved myelination in the hippocampus. Our findings could help elucidate the pathophysiology of MDD. In addition, our results indicate that LXR agonists may be useful in future strategies for antidepressant treatment.

## Data Availability

The raw data supporting the conclusions of this article will be made available by the authors, without undue reservation.
